# Correlation between antibody response against porcine epidemic diarrhea virus in sows and their offspring under field conditions

**DOI:** 10.14202/vetworld.2021.1689-1694

**Published:** 2021-06-29

**Authors:** Prapassorn Boonsoongnern, Orawan Boodde, Wilairat Chumsing, Manakorn Sukmak, Pichai Jirawattanapong, Nattavut Ratanavanichrojn, Alongkot Boonsoongnern

**Affiliations:** 1 Department of Anatomy, Faculty of Veterinary Medicine, Kasetsart University, Bangkok, Thailand; 2 Department of Farm Resources and Production Medicine, Faculty of Veterinary Medicine, Kasetsart University, Nakhon Pathom, Thailand

**Keywords:** antibody response, correlation, piglet, porcine epidemic diarrhea virus, sow

## Abstract

**Background and Aim:**

Thai pig farmers have suffered huge financial losses from porcine epidemic diarrhea (PED) since 2007. PED, caused by the PED virus (PEDV), leads to severe diarrhea, vomiting, and subsequent dehydration in suckling piglets. Lactogenic immunity derived from colostrum and milk is very important because immunoglobulins (Ig) cannot cross the placenta in pregnant sows. The aim of this study was to investigate the immunological correlation of the sample-to-positive (S/P) ratios of IgA and IgG against PEDV between colostrum, sow serum, and their piglet serum.

**Materials and Methods:**

A total of 43 sows were divided into three groups according to the experience of PEDV infection: Negative sow group (n=7) and treatment group (n=36, sows previously infected with PEDV). The treatment group was subdivided into two groups: Sows immunized with live-attenuated PEDV vaccine (n=15) and sows immunized with feedback (n=21) at 3 weeks before farrowing. The 7-day-old piglets (n=425) were obtained from negative sows (n=89), vaccinated sows (n=150), and feedback sows (n=275). Colostrum, sow serum, and their piglet serum were collected and analyzed for S/P ratios of their IgA and IgG levels against PEDV using an enzyme-linked immunosorbent assay.

**Results:**

The piglets from sows immunized with live-attenuated PEDV vaccine had a higher S/P ratio of IgG against PEDV (p<0.001), whereas the piglets from the feedback group had a higher S/P ratio of IgA against PEDV (p<0.001) compared with piglets from the negative sows. In addition, the S/P ratios of PEDV-specific IgA and IgG between sow serum and colostrum showed a positive correlation (Pearson’s coefficient r=0.61 and 0.75, respectively). Both S/P ratios of PEDV-specific IgA and IgG in sow serum and colostrum had a positive correlation to those in piglet serum.

**Conclusion:**

Overall, this study suggested that pregnant sows immunized with the live-attenuated vaccine against PEDV and feedback may provide maternal immunity against PEDV to their offspring.

## Introduction

Porcine epidemic diarrhea (PED), an enteric disease, is caused by the PED virus (PEDV). PEDV, an enveloped, single-stranded RNA virus belongs to the order Nidovirales, family Coronaviridae, subfamily Coronavirinae, and genus Alphacoronavirus [1,2]. PED was first identified in Europe and has since been reported worldwide. PED outbreaks have spread throughout Thailand since 2007 [3]. PEDV infects all ages of pigs through a fecal-oral transmission with the most severe impact on suckling piglets with clinical signs of diarrhea, vomiting, and dehydration associated with high mortality [3-6]. Moreover, sows and gilts infected with PEDV have shown reduced reproductive performance, such as a decreased farrowing rate and number of piglets born per litter and an increased abortion rate, return to estrus rate, and percentage of mummified fetuses per litter [7].

The piglets have lactogenic immunity because their sows have an epitheliochorial placenta that cannot transfer maternal immunity during gestation [8]. Thus, newborn piglets can be protected against PEDV by maternal immunity present in the colostrum and milk of their immunized PEDV sow. Hence, maternal vaccination before parturition can generate a PEDV humoral immune response and passively transfer protection antibody in milk by the gut-mammary gland (MG)-secretory immunoglobulin A (sIgA) axis in neonatal piglets [9]. Furthermore, feedback (feeding the intestine of piglets infected with PEDV) is routinely used to stabilize herd immunity to all sows in the outbreak herd. For example, sows in an outbreak herd that was immunized with feedback could produce healthy liveborn pigs 3-4 weeks after exposure [10]. Therefore, gestating sows and neonatal suckling piglets are immunized with feedback and PEDV vaccine for prevention and control of infection by inducing antibodies against PEDV in Thai pig farms.

The aim of this study was to investigate the immunological correlation of the sample-to-positive (S/P) ratios of IgA and IgG against PEDV between colostrum, sow serum, and their piglet serum.

## Materials and Methods

### Ethical approval

All experiments on animals were carried out in accordance with the Ethical Guidelines of Laboratory Animals in Research, National Research Council, and were approved by the Animal Care and Use Committee, Kasetsart University (ACKU 03358), Bangkok, Thailand.

### Study period and location

The study was conducted from February to July 2019 at the three different commercial farms located in Suphan Buri, Chonburi, and Ratchaburi provinces, Thailand.

### Virus and cells

PEDV, TRANG/37 strain, Thailand (accession number: MN379926) was isolated from infected field fecal sample and was propagated in Vero cells. Vero cells were maintained in Minimum Essential Medium supplemented with 5% fetal bovine serum containing 100 units/mL penicillin/mL, 100 mg streptomycin/mL, and 5% trypsin at 37°C with 5% CO_2_. Thereafter, 150 passages were used for PEDV live-attenuated vaccine preparation. Finally, 10% of a commercial adjuvant Montanide™ Gel 01 (Seppic, Seoul, Korea) was added into a PEDV live-attenuated vaccine that contained 1×10^5^ TCID/mL PEDV.

### Feedback

Feedback was obtained from intestinal samples from PEDV-infected 3-day-old piglets. After the first detection of PEDV by PEDV antigens test kit (BioNote, Republic of Korea), intestinal samples were confirmed by real-time polymerase chain reaction using a previously described method [11]. An infected piglet was humanely euthanized following the standard protocol. The intestine was homogenized; 5 L distilled water and then 5 mL 4% gentamicin and 200 g milk replacer were added. This amount of intestinal homogenate could be fed to approximately 50 sows that were orally administered 100 mL homogenate each.

### Experimental design

#### Sows

Forty-three sows within parities 1, 3, and 5 were selected from three different Thai pig farms; of them, one farm was never infected with PEDV, and the rest two pig farms had experienced PEDV infection. The 43 sows were divided into three groups: (a) Seven sows from the PEDV-free farm that received neither PEDV vaccine nor feedback as a negative control group, (b) 15 sows with previously infected PEDV that received the live-attenuated vaccine for PEDV by intramuscular injection, and (c) 21 sows with previously infected PEDV that was immunized with feedback. Vaccination and feedback were performed 3 weeks before farrowing.

### Piglets

Eighty-nine piglets from the negative sows were used as a control group. Four hundred and twenty-five piglets from immunized sows consisted of 150 piglets from PEDV-vaccinated sows and 275 piglets that received feedback from their sows.

### Sample collection

Blood samples were collected from sows at 7 days before farrowing and from their 7-day-old piglets from the jugular veins into sterile Vacutainer tubes (BD Vacutainer^®^ Blood Collection Tubes, Franklin Lakes, NJ, USA). The sera were separated using centrifugation at 1500× *g* for 15 min at room temperature (25°C). All samples were preserved at –80°C until tested.

Colostrum (volume 1 mL) was collected from the first front teats of sows within 1 h of farrowing. Samples were investigated after centrifugation at 13,000× *g* for 15 min at 4°C to remove fat and debris. All samples were kept at –80°C until tested.

### Indirect enzyme-linked immunosorbent assay (ELISA)

Anti-PEDV IgG and IgA in serum and colostrum samples were detected using ELISA. Briefly, PEDV was propagated on Vero cells and was harvested using centrifugation at 4000× *g* for 15 min at 4°C to remove cell debris. The whole viral purification was performed using ultracentrifugation at 13,000× *g* for 3 h at 4°C, then the pellet was collected. After washing twice with sterile phosphate-buffered saline (PBS) at pH 7.4, the pellet was resuspended in the same PBS at –80°C.

A total of 96-well polystyrene plates (Nalge Nunc International, USA) for cell culture were coated with 100 mL viral antigen in 0.5 M carbonate bicarbonate buffer (pH 9.6) at 4°C and were left overnight. After incubation, the plates were washed thrice with 0.01% Tween+PBS (PBST) and then blocked with 150 μL 5% skim milk in PBS at 37°C for 1 h. The plates were rewashed thrice with 0.01% PBST. Control and test samples (dilution 1:40) were added in duplicate (100 μL/well). Polystyrene plates were incubated at 37°C for 1 h and then washed 3 times with 0.01% PBST. Plates were incubated with 100 μL HRP-conjugated anti-swine IgG (KPL, MD, USA) in dilution (1:10,000 in 1% skim milk/PBS) or 100 μL HRP-conjugated anti-swine IgA (Thermo Fisher Scientific Inc., IL, USA) in dilution (1:4000 in 1% skim milk/PBS) at 37°C for 1 h. After washing, 100 μL of 3, 3׳ 5, 5׳-tetramethylbenzidine substrate (Kirkegaard and Perry Lab Inc., Gaithersburg, MD, USA) was added to each well plate. The reaction was stopped using 2 N H_2_SO_4_ and measured as optical density (OD) at 450 nm using an ELISA plate reader. The antibody response in sera and colostrum samples was represented as S/P ratios and represented as:

S/P ratio = (Sample OD–Negative control OD)/(Positive control OD–Negative control OD).

### Statistical analysis

The S/P ratios of anti-PEDV IgG and IgA in serum and colostrum were evaluated using Tukey’s honestly significant difference test with one-way analysis of variance in the R software version 3.3.2 (R Core Team, 2016). R: A language and environment for statistical computing. R Foundation for Statistical Computing, Vienna, Austria. URL https://www.R-project.org/. Data were expressed as mean±standard deviation. Linear mixed model regression with sow and piglet as random effects was employed to compare the levels of the S/P ratio of IgG and IgA against PEDV in piglets. Pearson’s correlation coefficient test was used to evaluate the correlation between (a) sow serum and colostrum samples, (b) sows and their piglet serum samples, and (c) sow colostrum samples and piglet serum samples. p≤0.05 was considered statistically significant.

## Results

**Figure-1:**
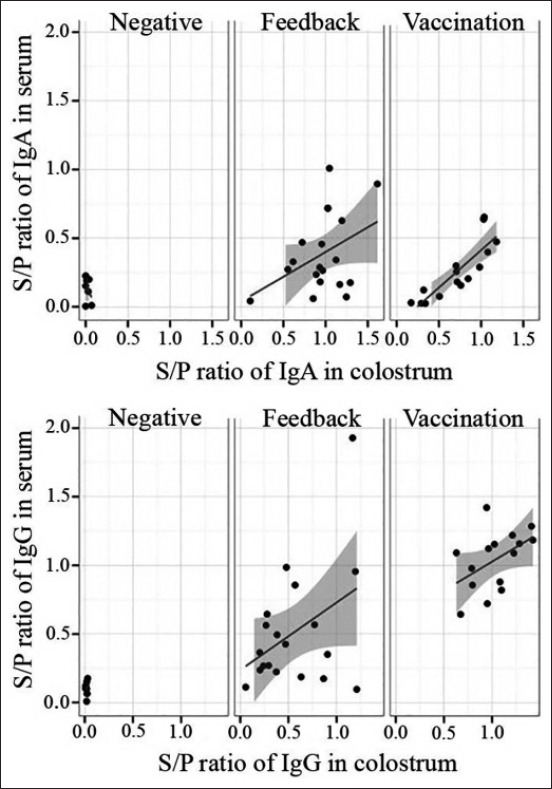
Comparison of antibody response to porcine epidemic diarrhea virus (PEDV) of sows with three different conditions: Negative, feedback, and vaccination. Analysis using Pearson’s correlation coefficient showed positive correlations of sample-to-positive ratios of both PEDV-specific immunoglobulin A and immunoglobulin G between sow serum and colostrum in sows immunized with a live-attenuated vaccine for PEDV and feedback.

**Figure-2:**
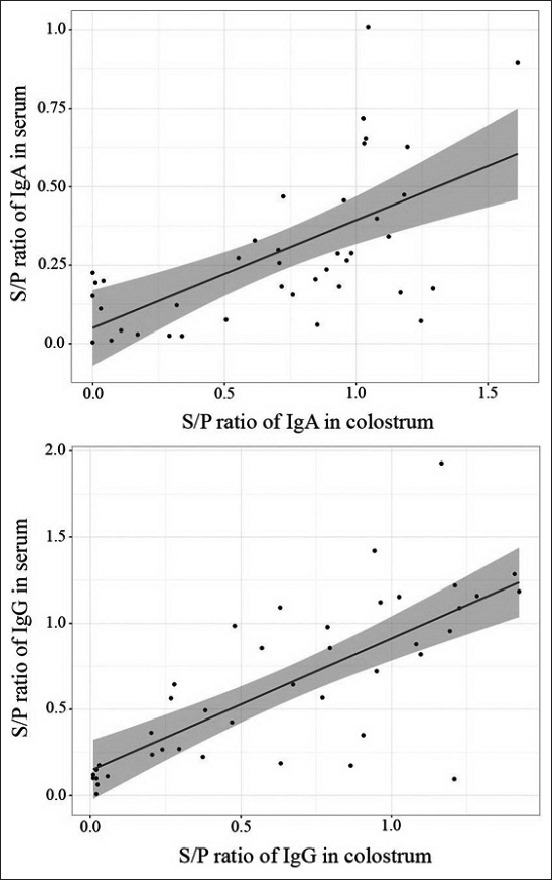
Pearson’s correlation coefficient of sample-to-positive ratio of porcine epidemic diarrhea virus-specific immunoglobulin A and immunoglobulin G between sow serum and colostrum shows a positive correlation (Pearson’s coefficient, r=0.61 and 0.75, respectively).c

Very low S/P ratios of both IgA and IgG against PEDV were detected in sow serum and colostrum of the negative group, whereas the immunized sows with feedback and vaccination showed higher S/P ratios of both PEDV-specific IgA and IgG compared to the negative group. The sows immunized with live-attenuated PEDV vaccine showed greater S/P ratios of PEDV-specific IgG in serum and colostrum, while sows immunized with feedback showed higher S/P ratios of PEDV-specific IgA in serum and colostrum (Figure-1). The S/P ratios of PEDV-specific IgA and IgG in sow serum showed a correlation with those in colostrum (Pearson’s r=0.61 and 0.75, respectively) (Figure-2).

**Figure-3:**
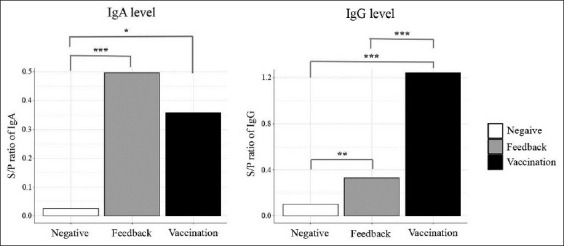
Sample-to-positive (sample-to-positive) ratios of porcine epidemic diarrhea virus (PEDV)-specific immunoglobulin A and immunoglobulin G of piglets obtained from sows with different conditions: Negative, feedback, and vaccination. The S/P ratios of immunoglobulin G against PEDV demonstrate significant differences between groups. The S/P ratios of immunoglobulin A against PEDV are significantly different between piglets from immunized sows compared with the control group, but there is no difference in the S/P ratios between piglets from differently immunized sows *p<0.05, **p<0.01, and ***p<0.001.

**Figure-4:**
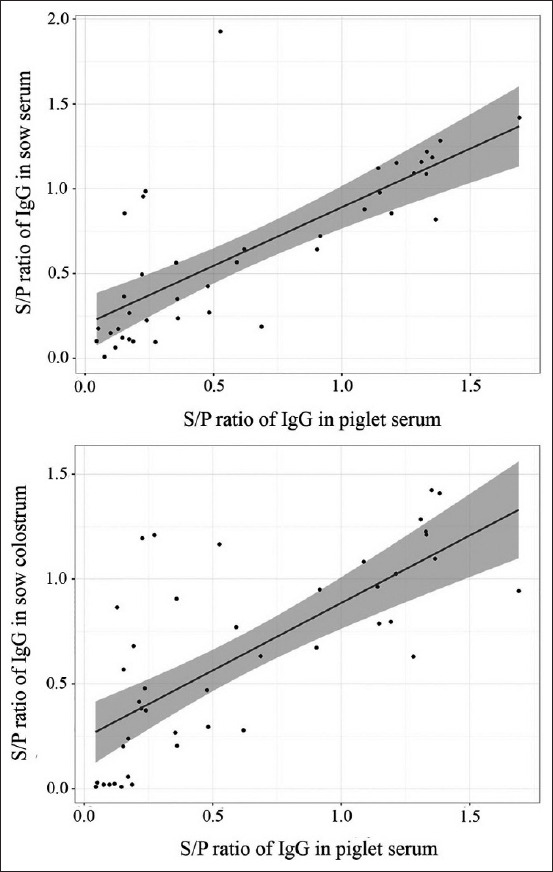
Positive correlation of sample-to-positive ratios of porcine epidemic diarrhea virus-specific immunoglobulin G between sow serum and piglet serum (Pearson’s r=0.74) and between colostrum and piglet serum (Pearson’s r=0.72).

**Figure-5:**
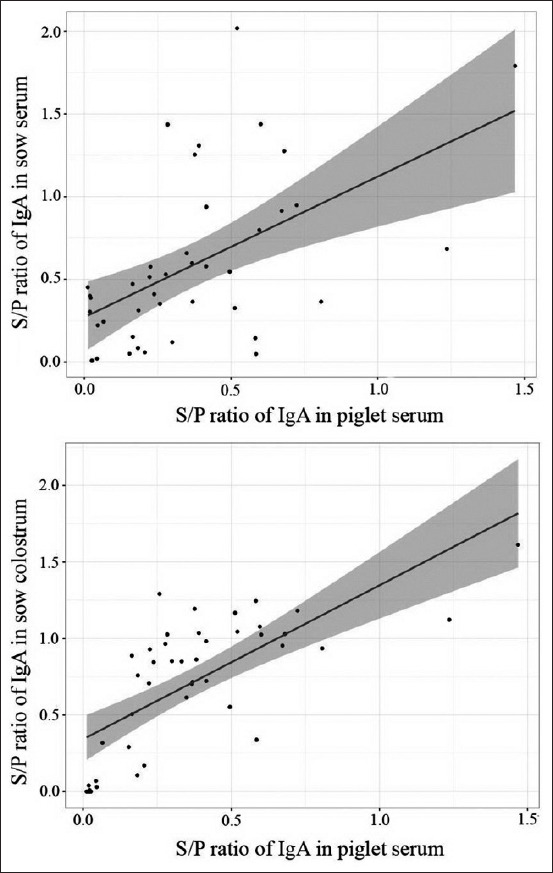
Positive correlation of sample to positive sample-to-positive ratios of porcine epidemic diarrhea virus-specific immunoglobulin A between sow serum and piglet serum (Pearson’s r=0.75) and between colostrum and piglet serum (Pearson’s r=0.54).

The piglets from vaccinated sows had the highest S/P ratios of IgG against PEDV in serum. In contrast, the piglets from immunized sows with feedback had higher S/P ratios of PEDV-specific IgA in serum (Figure-3). Furthermore, the S/P ratios of PEDV-specific IgG in piglet serum correlated positively with both sow serum (Pearson’s r=0.74) and colostrum (Pearson’s r=0.72) (Figure-4). Similarly, the S/P ratio of IgA against PEDV showed a positive correlation with sow serum (Pearson’s r=0.75) and colostrum (Pearson’s r=0.54) (Figure-5).

## Discussion

In this study, live-attenuated PEDV vaccine and feedback were used to immunize antibody levels against PEDV in sows. Subsequently, their piglets were expected to be protected against PEDV due to maternally derived immunity. According to several studies, antibody titers were generally high for a few weeks after vaccination; lactogenic immunity to PEDV induced during pregnancy through the gut-MG-sIgA axis facilitated piglet protection from PEDV [12,13]. The results indicated that sows immunized against PEDV with live-attenuated PEDV vaccine, and feedback showed increased S/P ratios of PEDV-specific IgG and IgA in serum and colostrum compared to the negative group (Figure-1). The PEDV-vaccinated sows had higher S/P ratios of IgG against PEDV in serum and colostrum, while feedback group sows had higher S/P ratios of IgA against PEDV in serum and colostrum (Figure-2). These results showed similarity to the findings by Paudel *et al*. [14], who demonstrated that vaccination against PEDV induced increased levels of IgG and IgA in sow serum and colostrum. The S/P ratios of IgG and IgA against PEDV in piglet serum trended similarly to sow serum and colostrum (Figures-4 and 5). These findings agreed with those by Bandrick *et al*. [15], who showed that the IgG and IgA levels in post-colostral piglets mimicked the levels of IgG and IgA in sow colostrums and the distribution pattern of IgG and IgA across colostrum and sow serum was similar. However, the presence of serum antibodies against gastroenteric pathogens is not always correlated with protection, as Song and Park [16] reported that the colostrum IgA concentration was a better marker of protection against PEDV infection. Findings in the current study showed higher S/P ratios of IgA against PEDV in both serum and colostrum in sows immunized with feedback because the feedback strongly stimulates mucosal immunity in the gut and triggers a quick response after immunization. However, the feedback has been shown to carry the risk of transmission of the contaminated viral and bacterial agents in the inoculum [10,17].

Even though the correlation of S/P ratios of IgA against PEDV between sow colostrum and piglet serum (Pearson’s r=0.54) was not as high as that between sow serum and piglet serum (Pearson’s r=0.75), the predominance of IgA may be transported from blood to milk for providing local passive immunity in the intestinal tracts of piglets [18]. IgA titers need to be monitored in colostrum and milk to ensure that piglets receive adequate passive immunity.

## Conclusion

The current study revealed that feedback immunization could improve an increase in PEDV-specific IgA in sow colostrum, whereas injectable attenuated PEDV vaccine-induced systemic immune response against PEDV. However, maternal-derived immunity can contribute to protecting their neonatal piglets against PEDV infection. Furthermore, it is suggested to conduct more accurate studies to further improve the strategies to manage and control the PEDV outbreak in pig farms.

## Authors’ Contributions

PB and AB: Designed the experiment. PB: Prepared manuscript and editing. AB: Contributed in writing, organization, and revision of the whole paper. AB and NR: Performed sample collection. OB, WC, and MS: Performed sample analysis. NR: Performed data collection. PJ: Performed statistical analysis. All authors read and approved the final manuscript.
